# Supplementary material for: Human xenobiotic metabolism proteins have full-length and split homologs in the gut microbiome
Source: G3 (Bethesda). 2025 Jun 7;15(9):jkaf131. doi: 10.1093/g3journal/jkaf131 (PMC12547972; doi:10.1093/g3journal/jkaf131)
Supplement: jkaf131_Supplementary_Data [file jkaf131_supplementary_data.zip › Supplemental_Material_Legends_G3-2024-405523.docx]

# Supplemental Figure and Table Legends

**Figure S1:** Trend in mitochondrial localization for full-length (orange) and split (teal) homologs, as a function of their distribution across gut species. Each dot represents all microbial homologs present in at least a certain number of gut microbial species (x-axis). The size of the dot corresponds to the total number of such microbial homologs. The y-axis shows what fraction of the human homologs are annotated as localizing to the mitochondrion. The baseline rate for all proteins is shown by the dashed line.

**Table S1:** Sensitivity analysis for proteins that had more split than full-length homologs in at least one of nine parameter sets.

**Table S2:** Sensitivity analysis for all proteins that had either at least one full-length or split homolog in at least one of nine parameter sets.

**Table S3:** GO term enrichment (biological process) for human proteins with more full-length than split homologs. Enrichments are shown for the full set of GO annotations (“all”), annotations that did not have the evidence code IEA (inferred from electronic annotation; “noIEA”), and annotations that had only low-throughput experimental evidence codes (“onlyExp”). Terms with adjusted p-values below 0.05 in any of the three sets are shown.

**Table S4:** GO term enrichment (biological process) for human proteins with more split than full-length homologs. Enrichments are shown for the full set of GO annotations (“all”), annotations that did not have the evidence code IEA (inferred from electronic annotation; “noIEA”), and annotations that had only low-throughput experimental evidence codes (“onlyExp”). Terms with adjusted p-values below 0.05 in any of the three sets are shown.

**Table S5**: Sensitivity analysis for full-length homolog GO enrichment analysis. Parameters tested are 50%/60%, 67%/70%, and 75%/80% for microbial and human coverage respectively.

**Table S6:** Sensitivity analysis for split-length homolog GO enrichment analysis. Parameters tested are 50%/60%, 67%/70%, and 75%/80% for microbial and human coverage respectively.

**Table S7:** List of species in which the UHGP-90 protein GUT_GENOME228173_01934, the top hit we found to *desE* (EDP16280.1), was found.

**Table S8:** Faith’s PD and richness (number of species) for each xenobiotic protein class at different alignment coverage cutoffs.

**Table S9:** Drugs metabolized by human proteins with mostly full-length homologs in the gut microbiome.
